# Correlates of absenteeism at work, school and social activities during menstruation: Evidence from the 2017/2018 Ghana Multiple Indicator Cluster Survey

**DOI:** 10.1371/journal.pone.0270848

**Published:** 2022-06-30

**Authors:** Stanley Kofi Alor, Emmanuel Anongeba Anaba, Philip Baba Adongo

**Affiliations:** 1 Department of Social and Behavioural Sciences, School of Public Health, University of Ghana, Accra, Ghana; 2 Department of Population, Family and Reproductive Health, School of Public Health, University of Ghana, Accra, Ghana; Cranfield University, UNITED KINGDOM

## Abstract

**Background:**

Menstruation is a biological process which is crucial for human reproduction. Menstruation is a source of absenteeism, yet the subject matter has not been well explored. This study aimed to assess the correlates of absenteeism at school, work and social activities during menstruation among Ghanaian women of reproductive age.

**Methods:**

This study was an analysis of secondary data from the 2017/18 Ghana Multiple Indicator Cluster Survey. Data were analysed using descriptive statistics, Chi-square and Binomial Logistic Regression with the aid of Stata/SE, version 16.

**Results:**

The majority of the participants were aged 25–49 years (63%), married/in union (55%) and resided in urban areas (52%). Nine in ten participants had access to privacy at home and 98% used menstrual materials during their last period. Eight in ten participants used disposable menstrual materials. Exactly 19% of the participants missed school, work or social activities during their last period. Participants who used disposable menstrual materials (AOR = 0.67; 95% CI: 0.52–0.85) were less likely to miss school, work or social activities during menstruation compared to those who used reusable menstrual materials.

**Conclusion:**

This study demonstrated that a significant minority of women in Ghana miss academic, economic or social activities during menstruation. Therefore, there is a need for effective interventions to help reduce menstruation-related absenteeism among women and girls in Ghana.

## Background

Menstruation is a natural biological process which is crucial for human reproduction. The onset of menstruation marks the beginning of the reproductive age in females [1]. Menstruation has been defined as “the natural bodily process of releasing blood and associated matter from the uterus through the vagina as part of the menstrual cycle” [2]. Statistics suggest that more than 1.8 billion persons worldwide menstruate every month [3]. Women and girls need adequate menstrual hygiene facilities (e.g. clean and safe menstrual materials, water, soap, disposal facilities and privacy) to help maintain optimal menstrual hygiene which can help prevent infections in the female reproductive tract [4,5].

Women and girls across the globe face many challenges during menstruation. These challenges range from lack of access to adequate and accurate information to lack of access to menstrual hygiene management facilities coupled with negative socio-cultural beliefs and norms surrounding menstruation [6–8]. For instance, about 500 million women and girls worldwide lack access to menstrual hygiene management facilities [9,10]. In Ghana, menstruation is still considered a taboo and restrictions (e.g. not entering the mosque, crossing rivers or cooking) are imposed on menstruators [11] which prevents them from performing their daily activities [12,13]. Also, some women experience menstrual pains which reduce their participation in activities [14,15].

Generally, menstruation is a source of absenteeism, hence it requires policy and research attention [16]. Studies have shown that the prevalence of menstruation-related work absenteeism ranges from 11% in Niger to 19% in Burkina-Faso. Menstruation-related school absenteeism also ranges from 15% in Niger to 23% in Nigeria [16]. In Ghana, evidence shows that the majority of adolescent girls miss school due to menstruation [17], with prevalence ranging from 27.5% to 95% [17–21]. For example, adolescent girls in the Upper East region missed between one to seven days of school per menstrual cycle [22]. Reasons cited for school absenteeism in Ghana include lack of access to sanitary pads/cloth, menstrual pains, fear of staining school uniform, teasing, odour and lack of privacy in school [17,19]. The risk factors associated with menstruation-related school absenteeism include cultural restrictions, older adolescent age [20], living in a rural area, menstrual pains, educational level and geographical region [23], while moderate parental income, positive attitude towards menstruation [20,23] and using disposable pads [24] were protective factors against menstruation-related school absenteeism.

The negative impact of absenteeism at work, school or social activities during menstruation cannot be overemphasized. It poses a threat to women’s and girls’ empowerment which can be achieved through participation in educational and economic activities [18]. Also, restrictions imposed on women during menstruation are violations of their fundamental human rights [25]. If these issues remain unaddressed, it may delay Ghana in achieving the Sustainable Development Goals (SDGs), especially goals that focus on promoting gender equality, inclusion in quality education, and good health and well-being [26].

In Ghana, menstruation-related absenteeism has received little research attention. Existing studies are skewed towards adolescent girls and school absenteeism [17,19] with little attention on adult women and absenteeism at work and social activities. The recent Ghana Multiple Indicator Cluster Survey (round six) collected data on menstruation-related behaviours among women of reproductive age, including the combined proportion of absenteeism at any of these activities (school, work or social activities) due to menstruation [27]. Yet, no study has explored the correlates of absenteeism at school, work or social (SWS) activities during menstruation among women in Ghana. Stakeholders can leverage the findings of this study to inform menstrual health policies and programmes. This will help Ghana to achieve SDGs three, four, five and six. The objective of this study was to assess the prevalence of menstruation-related absenteeism and associated factors among women of reproductive age, using nationally representative data.

## Methods

### Data source and study design

We analysed secondary data from the 2017/18 Ghana Multiple Indicator Cluster Survey (GMICS). The dataset was accessed through a formal request and approval to use the anonymised data was granted by UNICEF. The 2017/18 GMICS collected data on population and health indicators across all the previous ten regions of Ghana.

### Population and sampling

The 2010 Population and Housing Census list which was updated in 2017 was used as the sampling frame. The selection of participants was done in two stages, including, the selection of enumeration areas and the selection of households using a systematic random sampling technique, proportional to size. A total number of 12,886 households were interviewed. Women in the selected households who were between the ages of 15–49 years were eligible for the women’s survey. In all, 14,374 women were successfully interviewed by trained fieldworkers using Computer Assisted Personal Interviewing.

### Variables

The 2017/18 GMICS was the maiden MICS in the country to collect data on menstruation-related indicators. The focus of this study was on women of reproductive age (15–49 years). The dependent variable was absenteeism at school, work, or social activities during menstruation. This was measured with a single item (*due to your last menstruation*, *were there any social activities*, *school or work days that you did not attend*?), originally coded as ‘Yes’ = 1; ‘No’ = 2 and ‘do not know/not sure/no such activity’ = 8. This was recoded as ‘Yes’ = 1; ‘No’ = 0. ‘Do not know/not sure/no such activity’ was dropped from the dataset before the analysis.

The independent variables included age of respondent, level of education completed, religion and ethnicity of household head, marital status, parity, place of residence, region and disability status. Other independent variables were access to privacy at home, access to menstrual materials and type of menstrual materials, which were all categorical variables coded as ‘Yes’ = 1; ‘No’ = 2 and ‘Do not know’ = 8. Details about the 2017/18 GMICS are provided elsewhere [27].

### Ethical considerations

The 2017/18 MICS report stated that verbal consent was obtained from all the participants before questionnaires were administered. The Ghana Health Service Ethics Review Committee approved the study and the verbal consent. Informed consent was obtained from parents or legal guardians of participants below 18 years (minors). Moreover, participants were assured of voluntary participation, confidentiality, the anonymity of information and free will to withdraw from the interview at any point.

### Statistical analysis

Data were analysed with the aid of Stata/SE, version 16 (Stata- Corp, College Station, Texas, USA). We merged the household and the individual recode files. Besides, some of the independent variables were recoded. These include religion (Catholic = 1, Protestant = 2, Pentecostal = 3, Other Christians = 4, Islam = 5, Africa traditional = 6, No religion = 7), ethnicity (Akan = 1, Ga = 2, Ewe = 3, Mole Dbagni = 4, Others = 5) and age (15–24 = 1, 25–49 = 2). Also, no response and ‘do not know’ responses were dropped from the analytical dataset. In all, 12,795 women (weighted) were included in this study. The data were analysed in three levels, including univariate, bivariate and multivariable analyses. At the univariate level, frequency, percentage, mean and standard deviation were employed to analyze the data and summarised in tables. At the bivariate level, the Chi-square test was used to compute associations between the dependent and independent variables. At the multivariable level, Binomial Logistic Regression analysis was used to identify the salient predictors of menstruation-related absenteeism. Statistical significance was reported at the 0.05 significance level and odds ratios with a 95% confidence interval. The analysis took into consideration the complex survey design. To offset the challenges associated with oversampling, we adjusted for the sample weight.

## Results

### Socio-demographic characteristics of participants

The results demonstrated that the majority (63%) of the respondents were aged 25–49 years and 41% had junior high school education. Sixteen percent of the respondents were in the poorest wealth index, while 24% were in the richest wealth index. Three in ten respondents (31%) were from households where the head was a Pentecostal, while 49% of the household heads were Akan. Nine in ten respondents had no functional difficulties, 55% were married/in a union, while 52% lived in rural areas. More than half (67%) of the respondents were nulliparous. Concerning menstrual hygiene behaviours, 98% of the respondents used a menstrual material during their last period, 13% used reusable menstrual materials and 94% had access to a private place at home to change menstrual materials (Table 1). In addition, 19% of the respondents missed school, work or social activities (SWS) during their last menstrual period.

**Table 1:** Participant characteristics.

Characteristic	Missed SWS during menstruation		
	Total n (%)	No (%)	Yes (%)
**Region** Western Central Greater Accra Volta Eastern Ashanti Brong-Ahafo Northern Upper East Upper West	1291(10) 1269(10) 1673(13) 932(7) 1513(12) 3165(25) 1168(9) 1125(9) 369(3) 279(2)	91 87 90 80 91 71 66 81 86 77	9 13 10 20 9 29 34 19 14 23
**Age (years)** 15–24 25–49	4791 (37) 8004(63)	78 83	22 17
**Educational status** Pre-primary/no education Primary Junior high Senior high Higher	2216 (17) 2122(17) 5239(41) 2443(19) 775(6)	82 81 81 80 85	18 19 19 20 15
**Household wealth index** Poorest Second Middle Fourth Richest	2029(16) 2321(18) 2599(20) 2754(22) 3092(24)	82 76 81 82 83	18 24 19 18 17
**Religion of household head** Catholic Protestant Pentecostal Other Christians Islam Africa traditional No religion	1351(11) 1820(14) 4029(31) 2120(17) 2418(19) 510(4) 547(4)	75 85 82 84 78 79 80	25 15 18 16 22 21 20
**Ethnicity of household head** Akan Ga Ewe Mole Dbagni Others	6255(49) 1135(9) 1387(11) 1762(14) 2257(18)	81 86 83 78 79	19 14 17 22 21
**Disability status** Has functional difficulties Has no functional difficulties	987 (9) 10083(91)	81 82	19 18
**Marital status** Currently married/in a union Formerly married/in a union Never married/in a union	7014(55) 1215(9) 4566(36)	83 83 78	17 17 22
**Ever given birth** Yes No	8579(67) 4205 (33)	83 77	17 23
**Type of place of residence** Urban Rural	6598(52) 6197(48)	82 80	18 20
**Availability of private place at home** Yes No	11974 (94) 785(6)	80 89	20 11
**Used any menstrual materials** Yes No	12487(98) 269(2)	81 65	19 35
**Material reusable** Yes No	1607(13) 10857(87)	79 82	21 18

*p-value < 0.05.

It was found that one in five respondents aged 15–24 years did not participate in SWS activities during menstruation. Also, about one-third of the respondents in the Brong-Ahafo Region (34%) missed SWS activities during menstruation. Further, 35% of the respondents who did not use any menstrual material during their last period missed SWS activities. Moreover, 22% of women who have never married/in a union missed SWS during menstruation (Table 1).

### Predictors of absenteeism at SWS activities during menstruation

The crude analysis revealed that women between 15–24 years (COR = 1.35; 95% CI: 1.18–1.54) were more likely to miss SWS activities during menstruation compared to those aged 25–49 years. Also, women whose household heads were protestants (COR = 0.65; 95% CI: 0.44–0.96) were 35% less likely to miss SWS activities compared to those who professed Islamic religion. In addition, women whose household heads belonged to the Ga ethnic group (COR = 0.68; 95% CI: 0.50–0.94) had fewer odds of missing SWS activities compared to those belonging to the Akan ethnic group. Similarly, women who were never married (COR = 1.38; 95% CI: 1.21–1.57) were 1.38 times more likely to miss SWS activities compared to married women. Nulliparous women were 1.4 times more likely to miss SWS activities compared to their counterparts. Out of the previous ten regions, women in seven regions were less likely to miss SWS activities compared to women in the Ashanti region (p < 0.05).

The adjusted analysis showed that women in the Ashanti region had higher odds of missing SWS activities during menstruation compared to women in the Western, Central, Greater Accra, Volta, Eastern, Northern, Upper East and Upper West. Further, women who used disposable menstrual materials were 33% (AOR = 0.67; 95% CI: 0.52–0.85) less likely to miss SWS activities compared to those who use reusable materials. Also, women who had privacy at home to change menstrual materials were about three times more likely to miss SWS activities (AOR = 2.77; 95% CI:1.67–4.61) compared to their counterparts (Table 2).

**Table 2:** Binary Logistic Regression analysis of predictors of missing SWS activities during menstruation.

Covariates	COR (95% CI)	AOR (95% CI)
**Region** Western Central Greater Accra Volta Eastern Ashanti Brong-Ahafo Northern Upper East Upper West	0.23(0.16–0.32) * 0.36(0.26–0.49) * 0.27(0.19–0.37) * 0.59(0.42–0.83) * 0.24(0.16–0.36) * 1(ref) 1.30(0.96–1.75) 0.58(0.39–0.87) * 0.37(0.26–0.53) * 0.77(0.54–1.10)	0.19(0.13–0.27) * 0.28(0.19–0.41) * 0.24(0.16–0.35) * 0.42(0.29–0.62) * 0.21(0.14–0.32) * 1(ref) 1.14(0.84–1.55) 0.48(0.30–0.76) * 0.32(0.21–0.49) * 0.63(0.40–0.99) *
**Age (years)** 15–24 25–49	1.35(1.18–1.54) * 1(ref)	1.07(0.85–1.34) 1 (ref)
**Educational status** Pre-primary/none Primary Junior high Senior high Higher	1(ref) 1.09(0.86–1.39) 1.09(0.88–1.37) 1.13(0.87–1.46) 0.85(0.55–1.31)	1(ref) 1.20(0.89–1.62) 1.16(0.90–1.49) 1.10(0.82–1.48) 0.85(0.54–1.34)
**Household wealth index** Poorest Second Middle Fourth Richest	1(ref) 1.36(0.99–1.86) * 1.06(0.83–1.34) 0.92(0.73–1.16) 0.87(0.67–1.13)	1(ref) 1.39(1.04–1.85)* 1.12(0.84–1.51) 1.10(0.80–1.51) 1.17(0.82–1.68)
**Religion of household head** Catholic Protestant Pentecostal Other Christians Islam Africa traditional No religion	1.22(0.80–1.87) 0.65(0.44–0.96) * 0.83(0.58–1.20) 0.70(0.48–1.01) 1(ref) 0.98(0.61–1.57) 0.96(0.58–1.57)	1.32(0.92–1.88) 0.74(0.52–1.06) 0.99(0.71–1.37) 0.73(0.53–1.02) 1(ref) 1.07(0.72–1.59) 1.05(0.63–1.76)
**Ethnicity of household head** Akan Ga Ewe Mole Dbagni Others	1(ref) 0.68(0.50–0.94) * 0.85(0.66–1.10) 1.17(0.85–1.59) 1.17(0.92–1.48)	1(ref) 1.01(0.70–1.47) 1.11(0.82–1.49) 0.98(0.69–1.38) 0.93(0.70–1.23)
**Disability status** Has functional difficulties Has no functional difficulties	1(ref) 0.89(0.68–1.17)	1(ref) 0.87(0.67–1.13)
**Marital status** Currently married/in a union Formerly married/in a union Never married/in a union	1(ref) 0.94(0.73–1.21) 1.38(1.21–1.57) *	1(ref) 0.95(0.75–1.21) 1.21(0.92–1.60)
**Ever given birth** Yes No	1(ref) 1.41(1.25–1.58) *	1(ref) 1.32(0.99–1.77)
**Type of place of residence** Urban Rural	1(ref) 1.14(0.92–1.42)	1(ref) 1.18(0.92–1.52)
**Availability of private place at home** Yes No	2.04(1.38–3.02) 1(ref)	2.77(1.67–4.61) * 1(ref)
**Material reusable** Yes No	1(ref) 0.84(0.67–1.05)	1(ref) 0.67(0.52–0.85) *

*p-value < 0.05, COR: Crude odd ratio, AOR: Adjusted odd ratio, CI: Confidence interval.

## Discussion

Generally, the minority of the women missed school, work or social activities during menstruation [28–30]. Menstruation-related absenteeism was influenced by socioeconomic status, geographical region and type of menstrual material. We found that 19% of the women missed school, work or social activities during menstruation. This finding is consistent with findings in Burkina Faso, Niger and Nigeria, where 19%, 11% and 17% of women aged 15–49 years missed work due to menstruation. Moreover, 17%, 15% and 23% of girls aged 15–24 years in Burkina Faso, Niger and Nigeria respectively missed school due to menstruation [16]. These findings imply that menstruation poses a threat to women’s participation in academic, social and economic activities [16] which can affect their empowerment, inclusion in education and gender equality.

It was found that household wealth was associated with missing SWS activities during menstruation. For instance, women in the second wealth quintile and those living in the Ashanti region were more likely to miss SWS activities during menstruation. Our finding on socioeconomic status and menstruation-related absenteeism contradicts existing findings [16,22]. The Ashanti region is dominated by the Akans who believe that menstruation is taboo [31,32]. Gray literature shows that school girls were restricted from crossing the river *Offin* during menstruation. River *Offin* serves as a border between the Ashanti and Central regions of Ghana [33]. These socio-cultural norms and restrictions surrounding menstruation violate the rights of girls and women as well as widen the gender inequality gap and decrease girls’ inclusion in quality education.

Further, access to menstrual hygiene facilities reduces the odds of missing work, school or social activities during menstruation. Women who used disposable sanitary menstrual material were less likely to miss SWS activities. These findings have been confirmed by a previous study [17]. Girls who used reusable cloth pads to manage menstruation were more likely to miss school during menstruation [20]. A similar study in other West African countries revealed that women who used improvised menstrual materials had higher odds of missing work and school during menses [16]. These findings were expected because most women in Ghana use disposable sanitary pads, which are perceived to be comfortable and safe [20]. This implies that women who have access to disposable pads may have little fear of stains and odours, hence, they are more likely to participate in activities during menstruation.

### Implications and recommendations

These findings have implications for menstrual health policy, programming and research. The fact that one in five women missed activities during menstruation is a serious issue that requires the urgent attention of stakeholders. Ensuring gender equality and inclusion in quality education, eliminating all forms of discrimination against women, and ensuring women and girls’ empowerment and access to reproductive health rights are major targets of the SDGs. Promoting women and girls’ inclusion in education and socio-economic activities has numerous benefits, including reducing poverty, hunger, maternal mortality and child marriage [34,35]. These findings communicate that menstruation poses a threat to girls and women’s empowerment and inclusion in economic, academic and social activities. Therefore, stakeholders, including the Ministry of Gender, Children and Social Protection, Ministry of Education and Non-Governmental Organizations, who are interested in the health and wellbeing of women and girls should implement strategic programmes to help reduce menstruation-related absenteeism [36]. Moreover, interventions that aim at increasing women and girls’ participation in school and socio-economic activities during menstruation should prioritize women in the Ashanti region and those who use reusable menstrual materials. This study makes a significant contribution to the field of menstrual health research in sub-Saharan Africa. In Ghana, there is a paucity of literature on factors of menstruation-related absenteeism. This is the maiden study in Ghana to identify factors associated with menstruation-related absenteeism among women of reproductive age, using nationally representative data. This study highlights the scope of menstruation-related absenteeism in Ghana as well as the risk factors. Policymakers can leverage this empirical evidence to inform public health policies and programmes that will help increase women’s participation in activities during menstruation.

### Strengths and limitations

This is the maiden study to identify the salient factors associated with menstruation-related absenteeism among women of reproductive age in Ghana. Moreover, this study analysed nationally representative data, hence the findings can be generalized to the population. Notwithstanding, a major limitation of this study is the outcome. Combining absenteeism related to school, work and social activities during menstruation can be problematic, therefore, the findings should be interpreted with caution. It is recommended that future MICS should segregate menstruation-related absenteeism. In addition, association does not mean causation. Quantitative studies are unable to expose the many intricate views of participants regarding non-participation in socio-economic activities during menstruation, hence there is a need for qualitative studies.

## Conclusion

A significant minority of Ghanaian women miss academic, economic or social activities during menstruation. Menstruation-related absenteeism was associated with socioeconomic status, geographical region and type of menstrual material. Going forward, stakeholders should invest more resources in bridging the socio-economic inequalities. Menstrual hygiene interventions aimed at addressing menstruation-related absenteeism should prioritize women in the Ashanti region and those who use reusable menstrual materials.

## References

[1] WHO. World Health Organization. Adolescents in India. A Profile. (2003). Available at http://www.whoindia.org/LinkFiles/Adolescent_Health_and_Development_ (AHD) _UNFPA_Country_Report.pdf. 2003.

[2] UNICEF. Guidance for Monitoring Menstrual Health and Hygiene. New York: UNICEF, 2020.

[3] UNICEF. Guidance on Menstrual Health and Hygiene. New York: UNICEF 2019.

[4] Ademas A, Adane M, Sisay T, Kloos H, Eneyew B, Keleb A, et al. Does menstrual hygiene management and water, sanitation, and hygiene predict reproductive tract infections among reproductive women in urban areas in Ethiopia? PloS one. 2020;15(8):e0237696. doi: 10.1371/journal.pone.0237696 32822377 PMC7444535

[5] Torondel B, Sinha S, Mohanty JR, Swain T, Sahoo P, Panda B, et al. Association between unhygienic menstrual management practices and prevalence of lower reproductive tract infections: a hospital-based cross-sectional study in Odisha, India. BMC infectious diseases. 2018;18(1):473. doi: 10.1186/s12879-018-3384-2 30241498 PMC6150969

[6] Sommer M, Caruso BA, Sahin M, Calderon T, Cavill S, Mahon T, et al. A time for global action: addressing girls’ menstrual hygiene management needs in schools. PLoS medicine. 2016;13(2):e1001962. doi: 10.1371/journal.pmed.1001962 26908274 PMC4764363

[7] Phillips-Howard PA, Caruso B, Torondel B, Zulaika G, Sahin M, Sommer M. Menstrual hygiene management among adolescent schoolgirls in low-and middle-income countries: research priorities. Global health action. 2016;9(1):33032. doi: 10.3402/gha.v9.33032 27938648 PMC5148805

[8] Hennegan J, Dolan C, Wu M, Scott L, Montgomery P. Measuring the prevalence and impact of poor menstrual hygiene management: a quantitative survey of schoolgirls in rural Uganda. BMJ open. 2016;6(12). doi: 10.1136/bmjopen-2016-012596 28039290 PMC5223625

[9] World Bank. Its Time For Action: Investing in Menstrual Hygiene Management is to Invest in Human Capital 2019 [updated May 24, 2019; cited 2020 December 7]. Available from: https://www.worldbank.org/en/news/feature/2019/05/24/menstrual-hygiene-day-2019.

[10] Sommer M, Sahin M. Overcoming the taboo: advancing the global agenda for menstrual hygiene management for schoolgirls. American journal of public health. 2013;103(9):1556–9. doi: 10.2105/AJPH.2013.301374 23865645 PMC3780686

[11] Mohammed S, Larsen-Reindorf RE. Menstrual knowledge, sociocultural restrictions, and barriers to menstrual hygiene management in Ghana: Evidence from a multi-method survey among adolescent schoolgirls and schoolboys. Plos one. 2020;15(10):e0241106. doi: 10.1371/journal.pone.0241106 33091080 PMC7580927

[12] Thakur H, Aronsson A, Bansode S, Stalsby Lundborg C, Dalvie S, Faxelid E. Knowledge, practices, and restrictions related to menstruation among young women from low socioeconomic community in Mumbai, India. Frontiers in public health. 2014;2:72. doi: 10.3389/fpubh.2014.00072 25072044 PMC4080761

[13] Mohamed Y, Durrant K, Huggett C, Davis J, Macintyre A, Menu S, et al. A qualitative exploration of menstruation-related restrictive practices in Fiji, Solomon Islands and Papua New Guinea. PloS one. 2018;13(12):e0208224. doi: 10.1371/journal.pone.0208224 30507969 PMC6277107

[14] Acheampong K, Baffour-Awuah D, Ganu D, Appiah S, Pan X, Kaminga A, et al. Prevalence and Predictors of Dysmenorrhea, Its Effect, and Coping Mechanisms among Adolescents in Shai Osudoku District, Ghana. Obstetrics and Gynecology International. 2019;2019. 10.1155/2019/5834159 PMC6545782 31236112

[15] Aziato L, Dedey F, Clegg-Lamptey JNA. Dysmenorrhea management and coping among students in Ghana: A qualitative exploration. Journal of pediatric and adolescent gynecology. 2015;28(3):163–9. doi: 10.1016/j.jpag.2014.07.002 25823719

[16] Hennegan J, OlaOlorun FM, Oumarou S, Alzouma S, Guiella G, Omoluabi E, et al. School and work absenteeism due to menstruation in three West African countries: findings from PMA2020 surveys. Sexual and Reproductive Health Matters. 2021;29(1):1915940. doi: 10.1080/26410397.2021.1915940 33969811 PMC8118433

[17] Mohammed S, Larsen-Reindorf RE, Awal I. Menstrual hygiene management and school absenteeism among adolescents in Ghana: results from a school-based cross-sectional study in a rural community. International journal of reproductive medicine. 2020;2020. 10.1155/2020/6872491 PMC7204135 32411782

[18] UNICEF. Menstrual Hygiene. Challenges, Taboos and Impacts on Girls’ education. Ghana: UNICEF, 2016.

[19] Boakye-Yiadom A, Aladago DA, Beweleyir J, Mohammed HB, Salifu MF, Asaarik M. Assessing the Knowledge, Attitude and Practice of Menstrual Hygiene Management Among Junior High Schools Adolescent Females in the Yendi Municipality in the Northern Region of Ghana. European Scientific Journal, ESJ. 2018;14(36):467.

[20] Kumbeni MT, Ziba FA, Apenkwa J, Otupiri E. Prevalence and factors associated with menstruation-related school absenteeism among adolescent girls in rural northern Ghana. BMC Women’s Health. 2021;21(1):1–6. 34340667 10.1186/s12905-021-01418-x PMC8327434

[21] House S, Mahon T, Cavill S. Menstrual hygiene matters: a resource for improving menstrual hygiene around the world. Reproductive Health Matters. 2013;21(41):257–9.

[22] Kumbeni MT, Otupiri E, Ziba FA. Menstrual hygiene among adolescent girls in junior high schools in rural northern Ghana. The Pan African Medical Journal. 2020;37. doi: 10.11604/pamj.2020.37.190.19015 33447345 PMC7778209

[23] Davis J, Macintyre A, Odagiri M, Suriastini W, Cordova A, Huggett C, et al. Menstrual hygiene management and school absenteeism among adolescent students in Indonesia: evidence from a cross‐sectional school‐based survey. Tropical Medicine & International Health. 2018;23(12):1350–63. doi: 10.1111/tmi.13159 30286270

[24] Tegegne TK, Sisay MM. Menstrual hygiene management and school absenteeism among female adolescent students in Northeast Ethiopia. BMC public health. 2014;14(1):1–14. doi: 10.1186/1471-2458-14-1118 25355406 PMC4232635

[25] United Nations Population Fund. Period shame, misinformation linked to serious human rights concerns: UNPF; 2018 [cited 2021 March 22]. Available from: https://www.unfpa.org/news/period-shame-misinformation-linked-serious-human-rights-concerns.

[26] United Nations. Transforming our World:The 2030 Agenda for Sustainable Development. 2015.

[27] Ghana Statistical Service. Multiple Indicator Cluster Survey (MICS2017/18), Survey Findings Report. Accra: Ghana Statistical Service 2018.

[28] Naghma Rizvi Aga, Aga TSA. Misconceptions and Mismanagement of Menstruation among Adolescents Girls who do not attend School in Pakistan. 2016.

[29] Baumann SE, Lhaki P, Terry MA, Sommer M, Cutlip T, Merante M, et al. Beyond the Menstrual Shed: Exploring Caste/Ethnic and Religious Complexities of Menstrual Practices in Far-West Nepal. Women’s Reproductive Health. 2021;8(1):1–28. doi: 10.1080/23293691.2020.1861413

[30] Iliyasu Z, Galadanci HS, Abubakar IS, Ismail AO, Aliyu MHJJop, gynecology a. Menstrual patterns and gynecologic morbidity among university students in Kano, Nigeria. 2012;25(6):401–6. 10.1016/j.jpag.2012.08.006 23158770

[31] Agyekum K. Menstruation as a verbal taboo among the Akan of Ghana. Journal of Anthropological Research. 2002;58(3):367–87.

[32] Agana A-N, Prempeh C. Defiling the Church: The Impact of Mmusuo in Akan Conception. Transformation. 2020;37(1):3–17.

[33] Chronicle. Ghana traditional chiefs ban menstruating girls from crossing river. Chronicle 2018 January 12 2018.

[34] Birchall J. Early marriage, pregnancy and girl child school dropout. 2018.

[35] Bajracharya A, Psaki S, Sadiq M. Child marriage, adolescent pregnancy and school dropout in South Asia. Kathmandu; 2019.

[36] Bryn W. Taxes and duties for sanitary products in Africa. 2020.

